# Personalizing Personalized Medicine: The Pursuit of Optimal Thresholds in the Home OCT Artificial Intelligence Algorithm for Age-Related Macular Degeneration

**DOI:** 10.1016/j.xops.2025.101017

**Published:** 2025-11-26

**Authors:** Christina Y. Weng, Kevin J. Blinder, Edward F. Hall, William N. Rosenthal

**Affiliations:** Houston, Texas; St. Louis, Missouri; Rochester, New York; Overland Park, Kansas

This year marks 2 decades since the groundbreaking introduction of the first highly potent intravitreal anti-VEGF injections to treat neovascular age-related macular degeneration (nAMD).^1^, ^2^, ^3^ The emergence of these drugs solidified the early 2000s as a pivotal era that forever revolutionized the way we manage a once blinding eye condition. Fast forward to today, and the quest for even better nAMD outcomes continues. Leveraging therapeutics that offer longer durability is one way to potentially improve outcomes for patients with nAMD; individualizing actual treatment strategies is another. Both may be enhanced with the incorporation of remote OCT monitoring.^4^

In May 2024, the US Food and Drug Administration cleared the Notal Vision Home OCT device (SCANLY, Notal Vision, Inc), making it the first patient-operated, artificial intelligence–supported device for monitoring nAMD. This compact 16-pound machine enables patients to independently capture daily high-resolution volume scans (88 B-scans spaced 34 microns apart) that are securely stored on a portal accessible by designated medical personnel. The device works in tandem with the Notal OCT Analyzer (NOA), an artificial intelligence–based algorithm that identifies hyporeflective spaces and generates automated scan interpretations in volumetric terms. When the total hyporeflective volume (TRO)—indicating presumed fluid—exceeds a specified threshold, the system alerts the physician to take appropriate action, reflecting a personalized approach to disease management. Based on prior studies, a fluid threshold of 10 nL has commonly been used to distinguish between clinically meaningful retinal fluid and artifact.^5^^,^^6^ However, is it ideal to apply a universal threshold given the heterogeneity of disease activity? Or is it possible to further personalize this personalized approach? That pressing question is explored in this issue of *Ophthalmology Science*.

Leng et al^6^ conducted a post hoc analysis of the Home OCT Fluid Visualization Agreement Study that included 317 eyes of 180 participants who self-imaged daily with the Notal Vision Home OCT over a 5-week period.^7^ The study aimed to validate the artificial intelligence algorithm’s ability to detect TRO changes at different optimal fluid thresholds (OTs), using the assessment of 2 expert human graders as the ground truth for TRO stability or change. Three methods for identifying the OT were evaluated: (1) a personalized approach based on the reference change value to define a clinically significant change for that particular individual; (2) an optimized uniform approach that accounts for patient-specific TRO variability relative to a population-based reference data set; and (3) a uniform threshold of 10 volume units, equivalent to 10 nL of subretinal or intraretinal fluid. Their findings demonstrate that NOA was highly accurate in its determination of TRO change versus stability. Notably, the personalized approach exhibited higher sensitivity (99.1%) compared with the uniform approaches (94.4% for optimized uniform, 76.6% for 10 volume units uniform).

This analysis accomplishes 2 things. First, it corroborates existing evidence supporting the accuracy of the NOA algorithm.^5^^,^^7^, ^8^, ^9^ Second, it suggests that further refinement of the algorithm may improve its performance even more. The complexity of this refinement process should not be underestimated because our understanding of where optimal threshold levels should be set is still evolving. Choosing a fluid threshold hinges on a delicate balance of tradeoffs; setting it too high risks delayed recognition of fluid, whereas setting it too low can lead to false positives with undue visits and cost burden. Getting it just right will be crucial as this device moves toward commercialization. Both retina specialists and patients want to know that the possibility of missing true disease activity (false negative) is minimized, underscoring the importance of sensitivity measures. It is worth noting that although sensitivity and specificity are generally inversely correlated, there was minimal tradeoff observed with the personalized versus uniform approaches. In other words, despite higher sensitivity with the personalized approach, specificity remained comparable across all methods (89.4% for personalized, 89.4% for optimized uniform, and 94.7% for 10 volume units uniform), suggesting that OT personalization may improve the intrinsic performance of NOA.^6^

As the authors point out, individual variation in patient attention span, eye positioning and fixation, and even diurnal changes, may contribute to inherent anatomic fluctuations. Therefore, standardizing this noise at an individual (or even eye) level, rather than a population level, seems logical. Results of any post hoc study must be interpreted cautiously, but if these findings are substantiated, the next step would be to identify an efficient way of determining an individual’s personalized OT. Moreover, given the dynamic nature of nAMD and the learning curve associated with device use, the personalized OT may need to be periodically reassessed. This longitudinal study was based on 5 weeks of Home OCT data; a longer timespan may have yielded different results by encompassing more treatment cycles among the nAMD eyes. Additionally, nearly one-fourth of included eyes had dry age-related macular degeneration (AMD), and although there were 317 study eyes, they came from 180 participants, which likely mitigated the impact of individual-level variability. It would be worthwhile to analyze a larger cohort comprising exclusively eyes with nAMD because the changes in hyporeflectivity are undoubtedly more frequent and greater in magnitude than those observed with dry AMD.

Identifying optimal thresholds will require ongoing research efforts, ideally through prospective studies. One key prospective study currently underway is Diabetic Retinopathy Clinical Research Retina Network Protocol AO, a multicenter randomized clinical trial that will assign 600 eyes with treatment-naïve nAMD to receive faricimab on either a Home OCT-guided regimen or a treat-and-extend schedule that mirrors what the vast majority of retina specialists follow today.^10^^,^^11^ The coprimary outcomes will be the mean change in visual acuity and number of injections at the 2-year mark. This trial will provide invaluable insights into how Home OCT can be effectively integrated into clinical practice.

Previous studies have confirmed excellent usability of the Home OCT device, with 88% to 97.6% of patients able to obtain adequate-quality scans without assistance in <45 seconds.^5^^,^^8^^,^^9^ Adherence to scanning protocols, which is actively supported by Notal Vision, Inc, has been impressive with patients averaging >6 scans per week.^5^^,^^8^^,^^12^ Not only will Protocol AO fortify our understanding of device feasibility and long-term scanning compliance, but it will also enrich our knowledge about fluid thresholds that guide when participants should present for in-office assessments. In the Home OCT arm of Protocol AO, the fluid threshold is set to 10 nL. Hence, some eyes with stable levels of fluid below this threshold may be monitored without treatment, whereas eyes in the treat-and-extend arm will receive treatment for any detectable fluid. This design will allow comparison of outcomes arising from different degrees of fluid tolerance, a topic of considerable interest but with limited prospective data available.^13^ Secondary outcomes, including number of visits, development of macular atrophy, socioeconomic impact, and patient- and site-reported outcome measures, will also be assessed.

The importance of studies such as Protocol AO and the Home OCT Fluid Visualization Agreement Study becomes clearer when viewed in the broader context of Home OCT technology’s potential benefits. These can be classified into 4 categories:

(1) *Detect the development of exudative changes.* Patients with dry AMD face a 7% to 15% risk of complication by the wet form, and baseline visual acuity is a major predictor of long-term visual outcomes.^14^^,^^15^ Home OCT facilitates more frequent monitoring compared with standard of care, which may aid in earlier detection and treatment resulting in better outcomes; this will be tested in a parallel Protocol AO ancillary study that monitors fellow eyes with dry AMD for exudative changes.
(2) *Gauge response to treatment.* It is not uncommon for patients with nAMD to present with similar amounts of fluid on OCT at every clinic visit, which can fuel uncertainty about the patient’s response to the drug. Home OCT fluid volume trajectories map out quantitative fluid data and have illustrated the heterogeneity of treatment responses, with some eyes showing immediate, striking improvements, and others taking longer or never fully drying between visits. This information could help direct drug choice, especially with multiple options available today.
(3) *Guide and personalize treatment of nAMD.* To balance outcomes and treatment burden, most retina specialists employ a treat-and-extend strategy in treating nAMD.^10^ The unfortunate reality of treat-and extend, however, is that essentially every injection represents either overtreatment (if fluid is absent) or undertreatment (if fluid is present)—but this is the best we can do without the ability to assess intervisit disease activity. Through real-time monitoring, Home OCT could be transformative and bring us much closer to truly personalized care, where a patient receives an injection when and only when their fluid recurs.
(4) *Permit faster extension of treatment intervals.* Considering the limited evidence behind “loading” injections, along with the fact that eyes with no recurrent fluid after initiating therapy will still require almost a year to reach a quarterly or 16-week interval on treat-and-extend, one may wonder how often we administer injections that are not truly needed. With Home OCT, there are patients who have received a single injection at diagnosis and then immediately extended, sometimes going months before their next required treatment. This application of Home OCT will be especially appealing with longer-durability agents looming on the horizon (e.g., tyrosine kinase inhibitors and gene therapies), equipping patients and retina specialists with greater confidence in extending the time between visits while also prompting retreatment for those who warrant it sooner than anticipated.

Remote monitoring has advanced patient care across many fields, from continuous glucose monitoring in diabetes to cardiac monitoring for arrhythmias. In ophthalmology, however, our ability to leverage powerful technologies such as OCT remains limited by the need for in-clinic visits. Home OCT can change that. Practical considerations—such as workflow integration, reimbursement models, and medicolegal implications—still need to be addressed, but if the benefits of Home OCT are proven, the ophthalmic community will work to navigate these challenges. It was not that long ago when anti-VEGF injections ignited a paradigm shift in how we manage nAMD. Twenty years later, Home OCT could be on the verge of doing the same, paving the way for a future where earlier, more personalized interventions could meaningfully reshape patient outcomes.

Our ability to leverage powerful technologies such as OCT remains limited by the need for in-clinic visits. Home OCT can change that.
